# The effect of arm restriction on dynamic stability and upper-body responses to lateral loss of balance during walking: an observational study

**DOI:** 10.1098/rsos.241156

**Published:** 2024-12-11

**Authors:** Uri Rosenblum, Adi Lavi, Arielle G Fischer, Yisrael Parmet, Amir Haim, Shirley Handelzalts

**Affiliations:** ^1^ Department of Physical Therapy, Faculty of Health Sciences, Ben-Gurion University of the Negev, Beer Sheva, Israel; ^2^ Department of Health Sciences, Brunel University London, London, UK; ^3^ Department of Physical Therapy, Loewenstein Rehabilitation Medical Center, Ra’anana, Israel; ^4^ Department of Biomedical Engineering, Technion—Israel Institute of Technology, Haifa, Israel; ^5^ Department of Industrial Engineering and Management, Ben-Gurion University of the Negev, Beer Sheva, Israel; ^6^ Department of Orthopedic Rehabilitation, Loewenstein Rehabilitation Medical Center, Ra’anana, Israel; ^7^ Faculty of Medicine, Tel Aviv University, Tel Aviv, Israel

**Keywords:** walking perturbations, arm restriction, dynamic stability, perturbation-based balance training, fall prevention

## Abstract

When losing balance, upper-body movements serve as mechanical aids to regain stability. However, it remains unclear how these movements contribute to dynamic stability during recovery from a lateral loss of balance while walking with arm restriction. We aimed to (i) quantify the effect of arm restriction on gait stability and upper-body velocities and (ii) characterize upper-body kinematic strategies in response to lateral surface translations under different arm restriction conditions. Healthy adults were exposed to lateral surface translations while walking on a computerized treadmill under three conditions: ‘free arms’, ‘1-arm restricted’ and ‘2-arms restricted’. Dynamic stability and upper-body velocities for the first step after perturbation onset were extracted. We found decreased dynamic stability in the sagittal plane and increased trunk velocity in the ‘2-arms restricted’ condition compared with the ‘free arms’ condition. Head and trunk movements in the medio-lateral plane were in opposite directions in 44.31% of responses. Additionally, significant trunk velocities were observed in the opposite direction to the perturbation-induced loss of balance. Our results support the contribution of increased upper-body velocities to balance responses following arm-restricted walking perturbations and suggest that the ‘2-arms restricted’ condition may be utilized as a perturbation-based balance training, focusing on head and trunk responses.

## 1. Background

Reactive balance responses are critical for maintaining stability following a sudden loss of balance. While extensive research has delved into understanding lower-limb reactions to balance disruptions [1–5], the contribution of upper-body responses in restoring balance are not fully understood. Increasing the understanding of upper-body responses to balance perturbations is vital for identifying rehabilitation training targets for fall prevention.

To avoid falls, regulating the velocity of the upper body (i.e. arms, head and trunk) is crucial due to their combined mass, which constitutes about two-thirds of the total body mass [6], their height above the feet (i.e. base of support (BoS)) and their impact on the movement of the centre of mass (CoM) [7,8]. Additionally, stabilizing the head on the trunk is essential for balance control [9–11].

During unperturbed walking, trunk and arm motion has been shown to enhance stability by counteracting the lower body’s angular momentum [12–15]. Conversely, studies have shown that walking with arms held may improve stability by increasing trunk inertia, which limits CoM displacement [16,17]. When introducing perturbations during walking, the lack of arm motion (i.e. restriction) has affected gait parameters [16,18] and led to a slower return towards the normal gait pattern compared with walking with normal arm motion [16].

In response to perturbations, upper extremity strategies have been characterized as a protective strategy against injury, such as positioning the upper extremities to absorb impact energy with the ground or as a recovery strategy to stabilize the body by either reach-to-grasp or assist in counterbalancing and decelerating the falling CoM [17,19,20]. A recent systematic review [21] highlighted differences in upper extremity responses to different perturbation types (e.g. unexpected perturbations during standing and slips and trips during walking) between young and older adults. For example, directional differences in upper extremity responses were demonstrated: older adults tended to move their arms in the same direction as perturbation, while young adults tended to move their arms opposite to the direction of perturbation. These directional differences suggest an attempt to arrest the fall at impact in older adults (i.e. to brace against impact) and an attempt to restore an upright position by decreasing fall directed CoM displacement in young adults (i.e. to restore balance and prevent a fall) [22,23]. Other studies focusing on slips reported bilateral arm flexion to counter the backwards loss of balance [20] and to reduce trunk extension velocity, which aids in regaining balance [24].

While most studies have focused on slips that induce a backward loss of balance [20,24–28] or trips [22,29] that induce a forward loss of balance, in the real world losses of balance can occur in all directions [30–32]. In fact, the literature shows that medio-lateral (ML) loss of balance is more challenging to older adults compared with anterior–posterior (AP) loss of balance [33]. It is frequently associated with more stepping errors (e.g. leg collisions) [34,35], with altered first-step characteristics and postural movements of the trunk [35] and greater arm reactions in older adults [34,35]. Finally, balance reactions to ML loss of balance were found to be predictors of falls [36].

While various measures are used in the literature to assess dynamic stability during walking [37,38], here, we focus on the margin of stability (MoS), i.e. the distance between the extrapolated CoM (XcoM; see equation (3.1) in §3.2) and the border of the BoS [39], which has a more direct relationship with the probability of falling [40]. The MoS has been widely used to assess dynamic stability during both unperturbed and perturbed walking conditions [37,41]. Previous research has demonstrated that MoS is a key determinant in identifying balance outcomes (i.e. loss of balance) following perturbations [1,42,43]. Moreover, significant differences in MoS during reactive steps to perturbations have been observed between older adults with and without a history of falls [44], as well as between those with and without arms constrained [45]. Additionally, significant changes in MoS were reported in response to perturbation-based balance training [46].

Limited data exist regarding upper-body responses to lateral perturbations while walking. Additionally, the potential effects of restricting the upper extremity movements on dynamic stability (i.e. MoS) and upper-body reactive strategies in response to lateral perturbations have not been experimentally tested. This is particularly important given that humans are most dynamically unstable in the frontal plane [47–49] and that daily activities (e.g. talking/texting messages on the phone or carrying a bag while walking) or certain orthopaedic/neurological conditions (e.g. upper limb fractures, stroke) may impose limitations on upper extremity responses.

A previous study that employed an arm restriction approach to investigate upper-body responses to slips in young adults demonstrated a significantly higher rate of falls when both arms were restricted [25]. It was suggested that the increased fall frequency in the arm-restricted condition resulted from the inability to initiate an effective alternative strategy to control the CoM excursion and recover balance. This alternative strategy should be utilized by other body segments (e.g. trunk and head). Since walking introduces inertia in the AP direction, while ML surface translation introduces acceleration in the ML direction, we should find mixed directions of acceleration for the different body segments. However, this study did not assess body kinematics and upper-body segments’ movement strategies used to recover balance (e.g. whether the body segments’ velocity was in the same direction as the perturbation-induced fall or in the opposite direction), limiting our understanding of the underlying mechanisms. Therefore, the objectives of the current study were twofold: (i) to quantify the effect of arm restriction on dynamic stability and upper-body kinematics in response to lateral surface translations during gait, and (ii) to characterize upper-body kinematics and strategies in response to lateral surface translations under different arm-restriction conditions. We hypothesized that (i) greater arm restriction will lead to decreased dynamic stability and increased upper-body segment velocities to compensate for restricted arm movement, and (ii) head and trunk velocities will be greater in the ML direction to counteract the initial velocity in the direction of balance loss (i.e. the perturbation-induced velocity). Analysing how upper-body segments respond to perturbations during gait under varying arm-restriction conditions may enhance our understanding of the compensatory strategies individuals use to recover balance. This analysis can provide valuable insights into the roles of the trunk and head in maintaining stability, which are often underexplored compared with lower-body movements.

## 2. Methods

### 2.1. Participants

Fourteen healthy young adults (seven females) participated in the study, with a median age of 35 years (range: 31−39 years), height of 1.72 m (range: 1.62–1.86 m) and weight of 74.2 kg (range: 55–92 kg). All participants had no neurological, vestibular or orthopaedic conditions that may affect their balance and/or gait when participating in the study. All participants signed an informed consent form prior to participating in the study. The study was approved by the institution review board at Loewenstein Rehabilitation Medical Center (LRMC) (approval number LOE-14-0021).

### 2.2. Procedure

**Figure 1.**
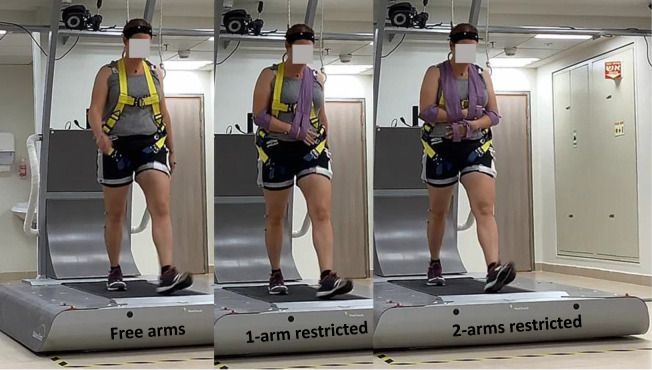
Experimental set-up. Participants walked on a computerized treadmill system with a horizontal movable platform (Balance Tutor, MediTouch Ltd, Tnuvot, Israel) at their preferred walking speed. Participants walked under three conditions, each lasting 120 s, in the following order: (i) unrestricted upper-body movement, ‘free arms’ condition (left); (ii) the dominant arm restricted with a sling, ‘1-arm restricted’ condition (centre); (iii) both arms restricted with slings, ‘2-arms restricted’ condition (right)’.

Assessments were conducted at the Gait Recovery Laboratory in LRMC. Participants walked on a computerized treadmill system with a horizontal movable platform (Balance Tutor, MediTouch Ltd, Tnuvot, Israel) at their preferred walking speed, determined from the average of two overground 10 m walk tests. Participants walked under three conditions, each lasting 120 s, in the following order: (i) unrestricted upper-body movement, ‘free arms’ condition; (ii) the dominant arm, determined by asking the participant which hand they use for writing, restricted with a sling, ‘1-arm restricted’ condition; (iii) both arms restricted with slings, ‘2-arms restricted’ condition (figure 1). In each condition, participants were exposed to four lateral surface translations (rightward, leftward, leftward and rightward) with a platform displacement of 12 cm, a velocity of 42 cm s^−1^ and an acceleration of 145 cm s^−2^ (these values are fixed within the perturbation system device, i.e. level 20). They were not informed about the timing or direction of the perturbations. Perturbations were introduced randomly every 30–45 s to reduce predictability. Participants were instructed to react naturally to prevent falling during the surface translations and to continue walking despite the perturbations. A safety harness attached to an overhead support was adjusted to prevent participants’ hands and knees from contacting the treadmill in case of a failed recovery. The harness did not support any body weight or restrict their movements during walking.

## 3. Kinematic data collection and analyses

Thirty-four reflective markers were strategically placed on anatomical landmarks in accordance with the Plug-In-Gait marker set (full body Plug-in-Gait model, Vicon Motion Systems, Oxford, UK). Three additional markers were affixed to the platform to synchronize platform movement data with kinematic data. Eight Vicon infrared motion capture cameras (Vicon Motion Systems, Oxford, UK) recorded kinematic data at a sampling rate of 120 Hz. Prior to analysis, motion data were preprocessed and analysed using Vicon Nexus software (v. 2.0) and checked for marker trajectory disruptions. Then, a three-dimensional model was generated using the software built-in function and the body CoM was extracted. The marker data were low-pass filtered using a zero-lag, fourth-order Butterworth filter with a cut-off frequency of 8 Hz. Gait events and parameters were computed using custom MATLAB scripts (v. R2022b; MathWorks, Natick, USA). Preprocessed data were used to identify the initial contact, defined as the local maxima of the heel marker position in the AP direction [50,51]. Gait spatio-temporal parameters (e.g. step length and width) were calculated from the marker data (see mean values in electronic supplementary material, table S1).

### 3.1. Kinematic outcome measures

All outcome measures were calculated for the first step after perturbation onset, as the main balance responses, particularly those involving the upper limbs, are most prominent in the early recovery phase following perturbations [22,52].

### 3.2. Margin of stability in the medio-lateral and anterior–posterior directions

To assess dynamic stability, the MoS in the AP and ML directions at the first initial contact after perturbation was calculated using equations (3.1) and (3.2), based on [39]


(3.1) $$XcoM=P_{CoM}+\left(V_{CoM}/\;\sqrt{\left(\frac{g}{l}\right)}\right),$$

where XcoM is the extrapolated CoM, *P*_CoM_ is the AP or ML component of the projection of the CoM on the ground. *V*_CoM_ is the CoM velocity in the respective direction subtracted by the perturbation platform velocity. The term √(g/l) presents the eigen frequency of an inverted pendulum system with a leg of length *l*.


(3.2) $$MoS=U_{max}-XcoM,$$

where MoS_x_ specifies the MoS in the AP or ML direction (from hereon the terms MoS_AP and MoS_ML will be used), *U*_max_ is the anterior or lateral boundary of the BoS defined as the toe marker in AP direction and lateral malleolus in the ML direction [51,53] and the XcoM is the position of the XcoM in the relevant direction.

### 3.3. Area under the curve of head and trunk velocities

To compute the velocity of the head and trunk, we first determined their positions. The head position was determined as the average of the four head markers in three-dimensional space. Trunk position was determined as the average position of the sternum, C7 and T10 markers in three-dimensional space. The first derivative of the preprocessed marker position of the trunk and the head were calculated to obtain velocity using equations (3.3) and (3.4)


(3.3) $$Trunk\;\;velocity\;=\;\frac{\Delta Trunk\;\;displacement}{\Delta Time}-Platform\;velocity$$

(3.4) $$Head\;velocity=\;\frac{\Delta Head\;displacement}{\Delta Time}-Platform\;velocity-\;Trunk\;velocity\;,$$

where platform velocity is the velocity of the platform during the perturbation.

Head and trunk velocity vectors were interpolated to 101 samples to normalize step time after perturbation to 100%. We used the *absolute* and *true* values of the head and trunk velocities to calculate the area under the curve (AUC): the absolute values were used to compute the velocity magnitude in the balance response (aim 1), and the true values were used to compute the dominant velocity direction (aim 2).

To highlight the preferred direction of the upper-body velocities in response to perturbations, we separated the ML and AP velocity components (aim 2). We also described the direction of the upper-body velocity in relation to the direction of the induced loss of balance (i.e. perturbation direction), such that velocities were increased in the direction of the loss of balance (positive values) or in the opposite direction to the loss of balance (negative values). For example, right surface translation induces a leftward loss of balance. If upper-body velocity in response to perturbation was towards the left, it was characterized as positive velocity in the direction of the induced loss of balance.

Perturbation direction was defined based on whether the surface translation was towards the participant’s dominant or non-dominant side. Finally, the perturbations were grouped into deciles according to the percentile of the gait cycle in which they were introduced to control for different perturbation timing.

### 3.4. Statistical analysis

All statistical analyses were performed using the ‘lme4’ package in R v. 4.2.2. Since participants’ characteristics were non-normally distributed (Shapiro–Wilk test), descriptive statistics are presented as median (range). Dependent variables comprised: MoS_AP, MoS_ML and the AUC of head and trunk velocities. Normality of variable distributions was assessed using distribution plots and the Shapiro–Wilk test on model residuals. Outliers were examined with the ‘check_outliers’ function using ‘*Z*-scores’, ‘ci’ and ‘iqr’ methods, identifying possible outliers for each outcome variable (see electronic supplementary material, table S2). As outliers did not affect the models, all samples were retained, and potential outliers were not removed. For aim 1, examining the effect of the ‘arm restriction condition’ on dynamic stability and upper-body velocities, *Z*-scores were standardized to ensure scaled coefficients across models. Then, we calculated four mixed-effect models, each representing one outcome variable, with ‘participants’ and ‘decile of gait cycle in which the perturbation was introduced’ as the *random* effects. Within-subject variables included: ‘arm restriction condition’ and ‘perturbation direction’ (surface translation towards the dominant versus non-dominant side). Post hoc analysis was conducted with Holm’s (sequential Bonferroni) method correction for multiple comparisons.

For aim 2, characterizing upper-body kinematics in response to lateral surface translations under different arm restriction conditions, two mixed-effect models (for the head and trunk) were used, with ‘participants’ and ‘decile of gait cycle in which the perturbation was introduced’ as random effects. Within-subject variables included ‘plane’ (AP versus ML), ‘arm restriction condition’ and ‘upper-body strategies’ (i.e. velocity magnitude in the direction or opposite direction of the loss of balance induced by the perturbation). Post hoc analysis with Holm’s (sequential Bonferroni) method correction for multiple comparisons was carried out.

## 4. Results

### 4.1. Participants

Participants’ characteristics are summarized in table 1. The dependent variables of the full dataset, arranged by participant, are summarized in electronic supplementary material, table S3. The initial dataset included 168 trials. Thirty trials had missing marker data that excluded them from the analyses, and eight trials in which the step after the perturbation was shorter than 10 kinematic data points were discarded (see electronic supplementary material, table S4 for summary of the discarded trials). Therefore, the final analysis included 130 observations of which 48 were in the ‘free arms’, 40 in the ‘1-arm restricted’ and 42 in the ‘2-arms restricted’ conditions.

**Table 1.** Participants’ characteristics. Abbreviations: M, male; F, female; R, right; L, left; BMI, body mass index.

participant	sex	age (years)	height (cm)	weight (kg)	BMI	walking speed (km h^−1^)	dominant side
01	F	35	1.62	57.2	21.79	5.2	R
02	M	36	1.74	77.1	25.46	5.1	R
03	M	37	1.86	76	21.96	5.7	R
04	F	34	1.64	75.7	28.14	5.2	R
05	F	38	1.68	57.5	20.37	4.8	R
06	M	39	1.78	78.6	24.8	4.6	L
07	F	37	1.64	55.2	20.52	4.4	R
08	M	34	1.84	92	27.17	6.4	R
09	F	35	1.75	78	25.46	4.3	L
10	M	33	1.82	72.7	21.94	4.6	L
11	F	34	1.62	72	27.43	4.3	R
12	M	33	1.74	82.7	27.31	5.4	R
13	F	38	1.66	55.6	20.17	4.6	R
14	M	31	1.71	67.6	23.11	3.6	R

### 4.2. Effect of arm restriction on gait stability and upper-body velocities

**Figure 2.**
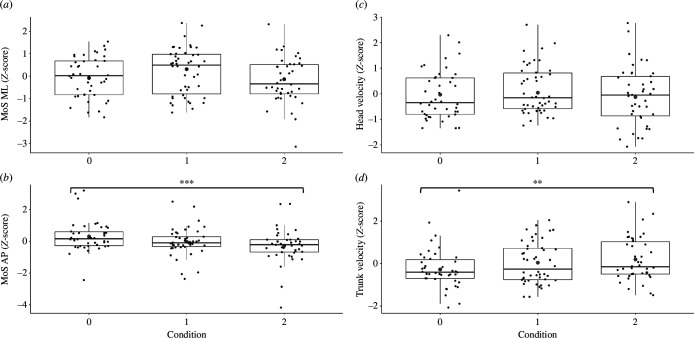
Boxplots of the effect of arm restriction condition on gait stability, i.e. MoS in ML plane (MoS_ML, (*a*)) and in AP plane (MoS_AP, (*b*)), head velocity (*c*) and trunk velocity (*d*). Condition 0, ‘free arms’; condition 1, ‘1-arm restricted’; condition 2, ‘2-arms restricted’. Small black dots represent actual data, while larger grey dots represent the mean predicted value by the model for each variable. The black solid horizontal line represents the median, upper whiskers represent the third quartile + 1.5 × interquartile range and lower whiskers represent the first quartile − 1.5 × interquartile range. **p < 0.01, ***p < 0.001.

Significant main effects of the ‘arm restriction condition’ were observed for MoS_AP (*F*_126,2_ = 6.55, *p* = 0.002) and trunk velocity (*F*_[126,2]_ = 4.25, *p* = 0.017). The ‘free arms’ condition exhibited significantly higher MoS_AP compared with the ‘2-arms restricted’ condition (odds ratio (OR) = −0.62, *t* = −3.62, *p* < 0.001; figure 2*b*); conversely, trunk velocity was significantly higher for ‘2-arms restricted’ condition compared with ‘free arms’ condition (OR = 0.48, *t* = 2.86, *p* = 0.005; figure 2*d*). No significant differences between arm restriction conditions were found for MoS_ML and head velocity (figure 2*a*,*c*).

### 4.3. Upper-body strategies

**Figure 3.**
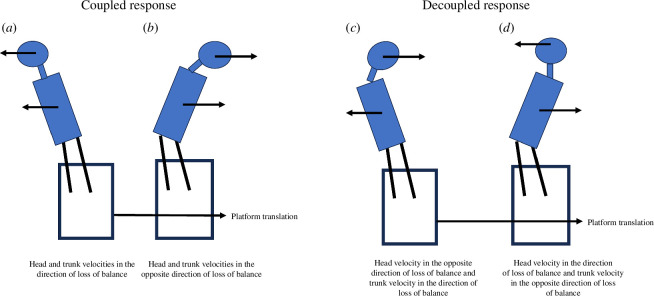
Upper-body strategies in response to lateral surface translations during walking. (*a*) Head and trunk velocities in the direction of loss of balance. (*b*) Head and trunk velocities in the opposite direction of loss of balance. (*c*) Head velocity in the opposite direction of loss of balance and trunk velocity in the direction of loss of balance. (*d*) Head velocity in the direction of loss of balance and trunk velocity in the opposite direction of loss of balance.

The movement of the head and trunk in the ML plane was either ‘coupled’ (i.e. the velocity of both the head and trunk was in the same direction, 36.15% of responses; figure 3*a*,*b*) or ‘decoupled’ (i.e. the velocity of the head and trunk was in opposite directions, 63.85% of responses; figure 3*c*,*d*). In the ‘coupled’ strategy, the upper-body velocities were either in the same direction as the direction of loss of balance (16.15% of responses; figure 3*a*) or in the opposite direction to the loss of balance (20% of responses; figure 3*b*). For the ‘decoupled’ strategy, in 37.69% of the responses, the head’s velocity was in the opposite direction to the loss of balance, while the trunk velocity was in the same direction as the loss of balance (figure 3*c*). In 26.15% of the responses, the velocity of the trunk was in the opposite direction to the loss of balance, while the velocity of the head was in the same direction as the loss of balance (figure 3*d*).

### 4.4. Upper-body kinematics

**Figure 4.**
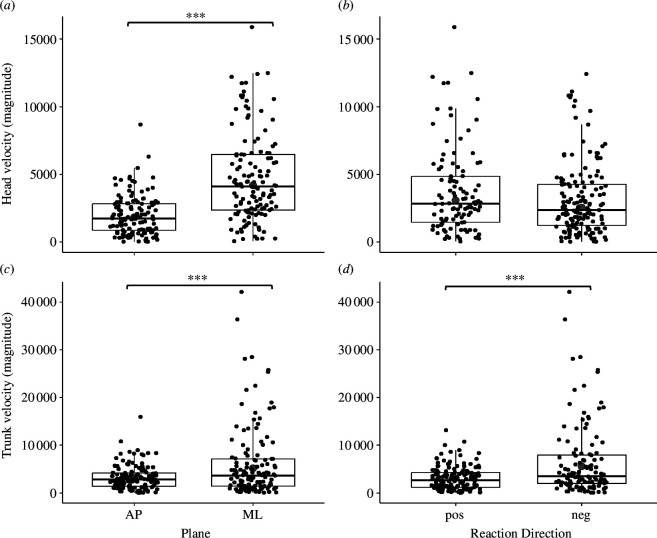
Boxplots of comparisons between upper-body velocities of the head (*a*,*b*) and trunk (*c*,*d*) in the AP versus ML planes and with (pos) or against (neg) the perturbation-induced body velocity direction. Small black dots represent actual data, while larger grey dots represent the mean predicted value by the model for each variable. The black solid horizontal line represents the median, upper whiskers represent the third quartile + 1.5 × interquartile range, and lower whiskers represent the first quartile − 1.5 × interquartile range. ***p < 0.001.

The models for the head and trunk showed significant main effects for ‘plane’ (AP versus ML; *F*_[1,255]_ = 105.02, *p* < 0.001; and *F*_[1,255]_ = 21.91, *p* < 0.001, respectively). Head and trunk had significantly greater velocities in the ML compared with AP plane (OR = 0.83, *t* = 8.89, *p* < 0.001 and OR = 0.48, *t* = 4.29, *p* < 0.001, respectively; figure 4*a,c*). We found significant main effect of ‘upper-body strategies’ for the trunk (*F*_[1,255]_ = 27.72, *p* < 0.001), indicating increased velocities in the opposite direction of the loss of balance (OR = −0.58, *t* = −4.64, *p* < 0.001; figure 4*d*). We did not find any other significant main effects (*p* > 0.06).

## 5. Discussion

In this study, we aimed to examine the effect of different arm restriction conditions on dynamic stability and upper-body velocities in response to lateral surface translations. We found that restricting the movements of two arms led to increased trunk velocity (but not head velocity) in comparison with ‘free arms’ condition and decreased MoS_AP (but not MoS_ML), partially supporting our hypothesis that greater arm restriction would result in decreased MoS and increased upper-body velocities. Increased head and trunk velocities were observed predominantly in the ML direction compared with the AP direction. While the increased trunk velocities in the ML direction were in the direction opposite to the loss of balance, the increased head velocities in the ML direction did not exhibit a preferred direction. This partially supports our hypothesis that upper-body velocities would be greater in the ML direction compared with the AP direction to counteract the loss of balance. Finally, we found that the upper-body strategy is a complex combination of head and trunk velocity that could be in the same direction (what we termed ‘coupled’ strategy) or in opposing directions (what we termed ‘decoupled’ strategy).

### 5.1. Effect of arm restriction on dynamic stability and upper-body velocities

Our results regarding the effect of arm restriction on dynamic stability and upper-body velocities in response to lateral surface translations during walking are consistent with previous literature. These studies show a significant effect of arm restriction on dynamic stability in response to balance perturbations during walking [16,54] and standing [55]. Previous research has shown a higher incidence of falls in the ‘arms restricted’ condition compared with the ‘free arms’ condition in response to a slip [54]. Another study demonstrated reduced gait stability when the arm swing was restricted [16]. Our findings further support these observations by showing a significant effect of 2-arm restriction on reduction in dynamic stability in AP direction and increased trunk velocity, predominantly in the ML direction, against the direction of the perturbation-induced fall. While the decrease in sagittal plane stability in the ‘2-arms restricted’ condition compared with the ‘free-arm’ condition cannot be attributed to changes in step strategies, as no significant differences in step length were found in the ‘2-arms restricted’ condition (electronic supplementary material, table S1 and figure S1), it may be related to the increased trunk velocity observed in the ‘2-arms restricted’ condition. While most of the trunk velocity was in the ML direction (figure 4), there was still a significant AP velocity component, which could contribute to reduced stability in the AP direction. Our results suggest that young, healthy adults can successfully compensate for their inability to react with their arms by increasing their trunk velocity to oppose the loss of balance and use it as a mechanism to control their CoM velocity. Trunk velocity seems to be the prime mechanism as there were no significant changes in MoS_ML. This raises the question of whether older adults or individuals with neurological disorders would demonstrate a similar strategy and whether a lower ‘restriction threshold’ that evokes a compensatory response of increased head and trunk velocities will be found. Further research is needed to explore these questions. Furthermore, the trend of increased velocities with increased difficulty level suggests that arm restriction might be useful as a methodology to focus perturbation-based balance training on trunk responses during walking.

Previous research has shown that trunk responses in the initial phase following lateral perturbations are affected by the magnitude of the perturbation, with larger perturbations resulting in greater deviations from steady-state walking. The perturbation magnitude used in our study was similar to the large-magnitude perturbations in [8]. Although we observed increased trunk velocities in the 2-arm restriction condition, we did not find significant differences in step width or step length across the different arm restriction conditions (electronic supplementary material, table S1 and electronic supplementary material, figure S1). While larger perturbations typically lead to changes in step responses [40] to maintain stability by placing the foot outward of the XcoM and increasing the MoS (i.e. dynamic balance) [56], the absence of such changes in our study could be attributed to the fact that our participants were young adults who may have relied more on trunk adjustments rather than stepping strategy.

Other possible mechanisms, which were not the focus of this work, may have also contributed to the lack of significant change in MoS_ML. Different strategies, such as the ankle and hip strategies, can be employed to maintain stability in response to perturbations during walking, together with the stepping strategy [57]. These strategies are influenced by the direction, timing and magnitude of the perturbation [8,57]. The ankle strategy involves shifting the centre of pressure laterally to control the CoM displacement. Although this is the fastest response, it is constrained to about 2 cm [56]. The hip strategy is an impulse-like increase in ground reaction force, and it is most prominent during perturbations while walking relatively slowly, combined with the ankle strategy [58]. At higher walking velocities, the stepping strategy is typically added to the response [58].

### 5.2. Upper-body kinematics and strategies

Our results showed that the head and trunk velocities were significantly greater in the ML direction compared with the AP direction when controlling for the ‘arm restriction condition’ and ‘upper-body strategy’. Additionally, trunk velocity was associated with upper-body strategy demonstrating higher velocity in the direction opposite to the direction of loss of balance compared with the same direction as loss of balance.

The findings of two distinct upper-body strategies in response to lateral perturbations, namely in the direction of the perturbation-induced loss of balance direction or in the opposite direction to it, are in line with previous literature [21]. While previous studies demonstrated arm velocities in the direction of the loss of balance in older adults, we show for the first time this behaviour in young adults in the context of head and trunk kinematics. We assume that in young adults this behaviour of responding with the head and trunk in the same direction as the loss of balance without falling is made possible by compensation of other balance response mechanisms such as arm movements and stepping responses. Nevertheless, testing this assumption requires further study. Finally, our results suggest that the head and trunk, many times referred to as HAT in the literature, are most dominantly affected by the trunk movement. Moreover, the data suggests that the head and trunk should be studied separately in the context of recovery from perturbations during walking, since apparently, on some occasions, their velocities are in different directions, alluding to different calculations by the central nervous system eliciting separate motor programmes.

## 6. Limitations

Several methodological constraints should be acknowledged. First, the timing of perturbations relative to the gait cycle phase could not have been controlled for in the paradigm due to technical constraints; thus, perturbations were introduced in different phases during the gait cycle. To control for that, we included the timing of the perturbations defined by the disentitle of the gait cycle as a random effect variable in our models. Also, we made sure that we had at least 10 data points after the perturbation, which assured lower variation of the perturbation onset in relation to the gait cycle.

Second, the arm restriction conditions were introduced in the same order between participants with no randomization, which potentially caused fatigue in the restriction conditions. One would expect to find reduced upper-body velocities if fatigue was introduced; however, we did not observe that in the results. On the other hand, one may argue that fatigue may affect lower-limb responses, leading to increased upper-body velocities for compensation. However, in this case, we should have observed negative correlations between parameters of step length and width and the upper-body velocities (i.e. reduced MoS would associate with increased upper-body velocities), but this was not demonstrated (see electronic supplementary material, figure S1). The lack of condition randomization could also have affected our results, showing the ‘free arms’ condition exhibited significantly lower MoS_ML compared with the ‘1-arm restricted’ condition but not in the ‘2-arms restricted’ condition, which was introduced as the last condition. This might be a result of the participants getting used to having their hands restricted and learning of the task. Future research should ensure randomization of conditions to rule out this possibility. Finally, our study was based on healthy young adults, and thus our findings cannot be generalized to other populations such as older adults and people with movement disorders. Learning about the compensatory strategies in these populations requires a separate investigation.

## 7. Conclusions

Our findings highlight the pivotal role of increased upper-body velocities as a compensatory mechanism for maintaining balance control in response to lateral surface translations during walking with arm restriction. In the current study, we observed that young adults use their trunk movement as a primary mechanism to regain balance from lateral perturbations during walking. Importantly, our observations challenge the assumption that, in young adults, upper-body velocities invariably oppose the direction of balance loss (see figure 4). Additionally, the coupled velocities of the head and trunk in the direction of balance loss suggest that while increased upper-body velocity is employed to maintain balance, other mechanisms, such as widening the BoS, may also be involved. The interplay between these different mechanisms requires further research. Finally, our findings challenge the traditional approach of studying the head and trunk as a single unit (HAT). Our results suggest that these are two entities that are sometimes treated differently by the motor system, leading to dominant velocities in opposite directions.

## Data Availability

The raw datasets used and/or analysed during the current study are available from the corresponding author on request. A table summarizing individualized mean gait spatio-temporal parameters and a table summarizing the median values and interquartile range of individualized preprocessed data used for the analysis are provided in electronic supplementary material, tables S1 and S3 [59].
